# Generative Adversarial Network (GAN)-Based Autonomous Penetration Testing for Web Applications

**DOI:** 10.3390/s23188014

**Published:** 2023-09-21

**Authors:** Ankur Chowdhary, Kritshekhar Jha, Ming Zhao

**Affiliations:** 1 6sense Insights Inc., San Francisco, CA 94105, USA; 2 School of Computing and Augmented Intelligence, Arizona State University, Tempe, AZ 85281, USA; mingzhao@asu.edu

**Keywords:** autonomous pentesting, Wireless Sensor Network (WSN), Internet of Things (IoT), Generative Adversarial Network (GAN), reinforcement learning, Web Application Firewall (WAF)

## Abstract

The web application market has shown rapid growth in recent years. The expansion of Wireless Sensor Networks (WSNs) and the Internet of Things (IoT) has created new web-based communication and sensing frameworks. Current security research utilizes source code analysis and manual exploitation of web applications, to identify security vulnerabilities, such as Cross-Site Scripting (XSS) and SQL Injection, in these emerging fields. The attack samples generated as part of web application penetration testing on sensor networks can be easily blocked, using Web Application Firewalls (WAFs). In this research work, we propose an autonomous penetration testing framework that utilizes Generative Adversarial Networks (GANs). We overcome the limitations of vanilla GANs by using conditional sequence generation. This technique helps in identifying key features for XSS attacks. We trained a generative model based on attack labels and attack features. The attack features were identified using semantic tokenization, and the attack payloads were generated using conditional sequence GAN. The generated attack samples can be used to target web applications protected by WAFs in an automated manner. This model scales well on a large-scale web application platform, and it saves the significant effort invested in manual penetration testing.

## 1. Introduction

Penetration testing is a method of evaluating the security posture of a network, by launching controlled attacks against crucial network services and users. The goal is to identify and patch the security holes before an attacker discovers them. An attack typically starts by targeting edge sensor devices, and the attacker tries to exploit the known or unknown vulnerabilities present in the network services. The attacker can exploit a vulnerability, to obtain sensitive information or elevated privileges on a machine. The metric for measuring successful attacks is the number of vulnerabilities exploited and the cumulative impact on the network’s Confidentiality, Integrity, or Availability (CIA) as a direct result of successful exploitation [1]. The attack progression depends on the network setup. The attacker can target individual vulnerabilities in isolation if the vulnerabilities are not dependent on one other, i.e., the need to exploit one vulnerability before another. If the network is multi-hop and follows fine-grained access control principles, the penetration tester must compromise multiple vulnerabilities that are dependent on one other.

In the past few years, we have observed a rapid surge in web application tools, technologies, and libraries, like NodeJS, React, AngularJS, and Ruby on Rails [2], being deployed on edge devices, e.g.,the user interface of a smart camera. Naturally, with each web application framework, inherent security vulnerabilities are reported every year [3]. While security researchers invest much time identifying and reporting these vulnerabilities, they need help to keep up with web application vulnerability discoveries manually [4]. Attackers have also invested in using deceptive means for masquerading original attacks [5]. Moreover, the emergence of sophisticated attacks, such as Advanced Persistent Threats (APTs) [6], has increased the need for the identification of attack patterns beyond traditional signature-based attack detection.

The penetration testing market is expected to grow from USD 1718 M in 2020 to USD 4598 M by the year 2025, a compound annual growth rate (CAGR) of 21.8% [7], to address the continuously escalating security challenges. The web application market is expected to reach USD 10.44 B by 2027. Surveys, such as MIT Technology Review, have reported a 3.5 M cybersecurity workforce shortage in 2021 [8]. There is a significant demand to use artificial intelligence (AI)-enabled pentesting techniques to automate and continuously improve pentesting outcomes, which used to be handled by skilled pentesters, who could investigate vulnerabilities in a multi-stage approach. An AI-based detection mechanism for detecting deception attacks has been discussed by Pang et al. [5]. This is similar to the APT attacks that use alternate variations of known attack patterns to deceive the web application firewalls (WAFs). As a result, an AI-enabled penetration test in the real world is similar to addressing an AI planning problem. The reward model aims to obtain the highest possible reward for exploited vulnerabilities, bypassing the security mechanism to defend against the attack variants, e.g., hlWAFs and Intrusion Detection Systems (IDSs).

The computational and storage capacities of sensor networks, which typically struggle with resource constraints, have been significantly improved by the recent merger of cloud computing with WSNs [9]. Incorporation of the IoT in WSNs has even increased the attacks’ surface manifolds. Sensing devices in WSNs normally collect sensor data and transmit them, without much processing, directly to the sink node; however, in IoT networks, sensing devices are more intelligent than WSN nodes [10]. In both scenarios, they eventually leverage the distributed edge network, to store data on the cloud servers. Hence, this exposes another attack surface on the cloud-based databases in WSNs and IoT communication contexts. The Cross-Site Scripting (XSS) attack, Cross-Site Request Forgery (CSRF), and injection-based attacks are a few examples [10,11,12].

Smart manufacturing integrates various technologies, like sensors, the Industrial Internet of Things (IIoT), and Supervisory Control and Data Acquisition (SCADA) production [13]. The latest industry standards drive this—yet, due to the advent of different web technologies, they are exposed to web-based attacks, like XSS. The three-layered structure of the IoT, i.e., the perception layer, the network layer, and the application layer, introduces security issues at multiple layers [14]. Accessibility to data is over a broad spectrum of devices and platforms. Similar to traditional networks, the application layer’s vulnerability to attacks, which varies based on the particular IoT scenario, is the primary security concern, even in sensor networks; hence, there is a need to develop adaptive defense strategies to counter these attacks [12].

Generative Adversarial Network (GAN)-based approaches have been successfully applied to network IDSs in recent years. IDSGAN [15] generates adversarial malicious traffic to attack IDSs, by deceiving and evading detection. The research utilized functional and non-functional features from the NSL-KDD dataset [16], to train a GAN model to fool a BlackBox IDS. The generator was able to fool different detection algorithms, such as Support Vector Machine (SVM) [17], Naive-Bayes classifier [18], Multi-Layer Perceptron (MLP) [19], and Decision Tree (DT) [20], by generating variations of network and host-based attacks, such as User to Root (U2R), Remote to User (R2U), and DDoS attack. The GAN model used by IDSGAN is slow to train. While generating valid samples for fooling an IDS works well, this may not scale well for a web application framework protected by a WAF. The unconditional generative model has no control over the mode of data generation. Model conditioning using additional information allows more targeted data generation [21]. The conditioning is based on the class labels or some of the data. The generator *G* and discriminator *D*, two building blocks of a GAN, are conditioned on some extra information, such as a class label *y* or data modality.

GANs have proven to be an effective approach to generating continuous data, such as images [22]. However, using GANs for generating discrete data or attack sequences, such as XSS and SQLI attack payloads, is challenging. The reason for this inherent limitation is that generation starts with random sampling, followed by a deterministic transform on the model parameters. The gradient loss of *D* is used to guide *G* to change the generated value and make it more realistic. In the case of discrete token generation, the slight change approach makes limited sense, because there might be no token in the limited dictionary of the generator. Recent works on sequence generation, such as SeqGAN [23], overcome this limitation by modeling data generation as a stochastic policy in a Reinforcement Learning (RL) setting.

We considered the problem of generating attack payloads that can bypass the signature-based web defense mechanism. The GAN is provided with conditional information on the attack labels. This helps in generating high-quality attack samples. There are some research works that involve the use of fuzzy logic for generating attack samples. The Fuzzy Logic System (FLS), introduced by Shahriar et al. [24], utilizes input from different attack types, described as top threats in Open Worldwide Application Security Project (OWASP) web attacks, and risk assessment models, to generate attack payloads. These payloads can be tested against PHP-based applications, to check the security risk level of different applications. The tokens used in fuzzy logic are often at individual character level. Our framework uses semantic tokenization and a Byte Pair Encoding (BPE) [25] algorithm, to create better tokens, so as to generate logically correct attack sequences.

Moreover, the fuzzy logic scales poorly as the input size increases. The number of variations of discrete tokens can be exponential, in terms of token space. In our semantic tokenization approach, the tokens can map to a constant set of classes, such as tags, script parameters, function body, hyperlinks, etc. This makes the space complexity of the token generation method polynomial, in terms of the maximum length of sequence and the number of attack tokens. Thus, conditional sequencing scales well with an increase in input size, compared to a fuzzy-logic-based approach.

In this research, we utilized conditional sequence generation, to target web application firewalls protecting web applications against application layer attacks, such as XSS, SQL Injection, and Directory Traversal. The semantic knowledge from security experts encoded the data modality required for the attacks. The sequence generation process utilized this information for generating targeted attack payloads. We tested the generated attack payloads against open-source ModSecurity WAFs [26] and AWS WAFs [27]. The payloads generated by the conditional sequencing were able to bypass ModSecurity WAFs and AWS WAFs.

These payloads can help improve the attack signatures in the WAF ruleset. The key contributions of this work are as follows:

- Conditional sequence generation, by understanding the semantic structure of web attack payloads. The technique helps in improving the training efficiency of the generator and in generating valid attack signatures that can fool the discriminator.
- Evaluation of generated attack samples on production-grade WAFs. We used ModSecurity and AWS WAFs to test the quality of generated web attack samples. We observed that 8.0% of the attack samples targeting AWS WAF allowed listing and that up to 44% of the samples targeting AWS WAF block listing were able to bypass the rules in place for blocking web attacks.
- Generating a GAN-based synthetic attack dataset, by training a GAN model on real and fake attack samples. This synthetic data can help to train web application layer defensive devices, such WAFs, against sophisticated attacks, like APT.

## 2. Related Work

Web attacks, such as XSS, can lead to disruption of confidentiality and availability in a Cyber–Physical System (CPS). Duo et al. [1] modeled a CPS based on time-driven and event-driven cyber attacks. Penetration testing can be considered as an event-driven attack simulation, to detect vulnerabilities in a CPS. Alsaffar et al. [28] conducted a study of different types of XSS attacks, and they proposed a greedy algorithm for the detection of XSS vulnerabilities in web applications. The program only considered a static set of rules for detecting XSS attacks. An automated mechanism to conduct pentesting in a controlled manner is challenging. Several approaches have been used, to formulate pentesting as a planning problem. Lucangeli et al. [29] used Partially Descriptive Domain Modeling (PDDL)-based attack modeling. This approach was limited, since it assumed complete information about the attack states and actions. Attack planning has been modeled as a Partially Observable Markov Decision Process (POMDP) problem by Sarraute et al. [30]. This helps in incorporating uncertainty, such as non-deterministic actions. The POMDP modeling used in this work has been examined in limited experimental settings. As the environment becomes more complex (an increased number of exploits and machines), the runtime of the POMDP solver increases significantly. A reinforcement learning (RL)-based approach to automated pentesting has been considered in research works [31,32]. Schwartz et al. [31] formulated the problem using Markov Decision Process (MDP) modeling. The authors noted that an RL approach was scalable only in small-scale environments. Schwartz et al. [33] improved on their earlier work, by using a modified version of the POMDP. The authors incorporated the defender’s behavior as part of the response to pentesting activities within the model of autonomous pentesting. Ghanem et al. [32] used the POMDP modeling approach. Naturally, the time consumed to conduct pentesting on small-scale networks using this approach is of an order of hours. Tran et al. [34] used multi-agent RL for decomposing action space into smaller subsets, to help conduct pentesting at scale. Zhou et al. [35] used an improved Deep Q-Network (DQN) for addressing issues with sparse rewards, by improving the exploration ability of the neural network. Some other approaches that have been used for autonomous pentesting include using contingency planning to model the problem. Empirical evaluation was conducted in a simulated setting with known vulnerabilities.

Adversarial examples have been used for generating fake images with success. Adversarial networks, such as GANs, use the generative network to generate counterfeit images/samples that fool the discriminate model with a knowledge base of real data samples. GAN-based models have been used in cybersecurity operations, such as password cracking, intrusion detection, and XSS attack payload validation. PassGAN [36] uses a deep learning approach for password guessing. PassGAN uses training on 9.9 million unique leaked passwords, and using a GAN-based password cracking approach produced better password guesses than well-known tools, such as John the Ripper and HashCat. IDSGAN generates malicious traffic records, to attack IDSs by evading detection. The IDSGAN design classifies traffic into functional and non-functional features. The authors altered the non-functional features, to generate adversarial examples for different attack categories, e.g., retaining intrinsic (session-based) and time-based features for a DDoS attack, and modifying content and host-based features. An empirical evaluation showed a low detection rate against attack classification algorithms for the NSL-KDD dataset. Deep Convoluted GAN (DCGAN) has been used by Yang et al. [37], to deal with unbalanced network intrusion data. Zhang et al. [38] used a Monte Carlo Tree Search (MCTS)-based algorithm, to generate adversarial XSS attack samples. The research restricted attack sample modification using predefined rules and used a GAN to optimize the detector and to improve the attack detection rate.

There is a lack of robust attack datasets that can help detect sophisticated attacks, such as APTs  [6,39]. The use of deception-based attacks for some recent datasets, such as DAPT2020 [40] and Unraveled [41], targeted a general class of APT attacks, by simulating the threat vectors used in APT attacks. As the scale of web infrastructure and web technologies expands, it will become difficult for security researchers to generate real attack samples by using attack simulations. This research proposes complementing datasets such as DAPT2020 [40], and Unraveled [41], by generating fake attack data from real attack samples. GANs can create adversarial examples that mimic sophisticated attack techniques, as we have demonstrated in this research. By incorporating these examples into the WAF training data, it is possible to bolster a WAF’s resilience against evasion attempts and to improve its effectiveness against more advanced attacks.

## 3. Background

The process of penetration testing involves information gathering about the target, such as open ports, service version, Operating System (OS), and using the information to mount targeted attacks against a service. Several tools and techniques help in conducting penetration testing. One key issue in using tools is that the known vulnerabilities limit them. Most of these vulnerabilities have an associated Common Vulnerability Enumeration Identifier (CVE-ID) stored in a Common Vulnerability Scoring System (CVSS) [42]. Some vulnerabilities are left unidentified during the development life-cycle of a product. These vulnerabilities are known as zero-day attacks [43].

### 3.1. Web Application Attacks

There are several parts of a web application that can be targeted by web application attacks. A typical web application includes a web application protocol, e.g., HTTP/S, server-side functionality, the use of scripts or code to generate dynamic content, application design flaws, authentication, and a data storage mechanism used by the application. Some well-known web vulnerabilities include session hijacking, bypassing authentication, SQL injection attacks, and XSS  [44]. The XSS attacks involve using some aspect of the application’s behavior, to carry out malicious actions against users. These actions include logging user keystrokes, and masquerading user privileges, to carry out unintended actions.

**Figure 1:**
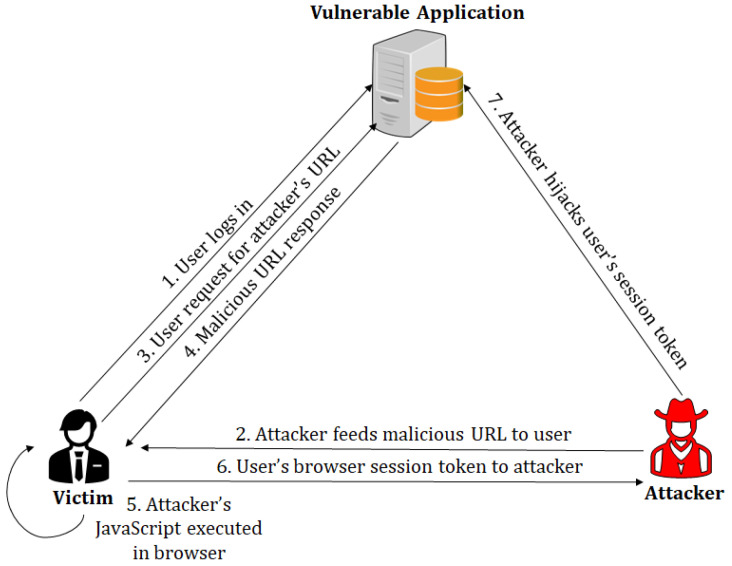
Cross-Site Scripting (XSS) vulnerability present in an application exploited by a remote attacker.

An example of how an attacker can capture the session token of an authenticated user has been provided in Figure 1. An authenticated user who logs into an application is issued a cookie—step 1. The attacker supplies a crafted URL to the user—step 2. The user requests the URL—step 3, and executes the malicious JavaScript returned by the attacker—steps 4 and 5. The malicious script requests the server owned by the attacker, with the user’s session token. In effect, the user’s captured token is supplied to the domain controlled by the attacker, and the user’s session is hijacked—steps 6 and 7. The payloads for such web-based attacks can also cause website defacement and other user actions, such as adding a new user with admin privilege (if the admin’s session has been hijacked).

#### Defense Mechanisms against Web Attacks

Modern servers and applications use several protection mechanisms to prevent web-based attacks. These techniques include (a) blocking the attacker’s input, based on an attack signature match, (b) input sanitation or encoding, and (c) truncating attack strings to a fixed length, to prevent attackers from injecting malicious scripts. Web applications exposed to the public internet make use of WAFs  [45] with these defense mechanisms, to filter and monitor application layer traffic. The attackers have also adapted to the defensive techniques employed by WAFs.

**Figure 2:**
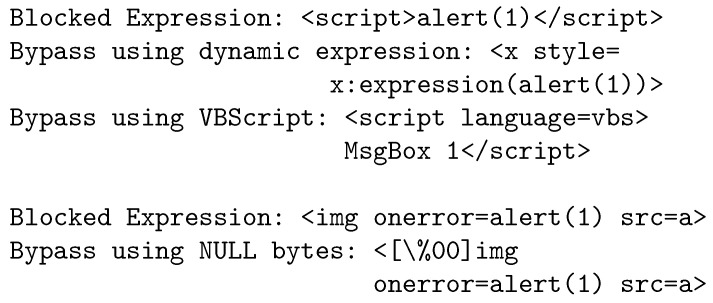
Expressions blocked by WAFs and corresponding bypass techniques.

Figure 2 shows expressions blocked by WAFs. The first attack vector uses script tags to insert the XSS payload. Modern WAFs can block expressions by signature matching, but a crafty attacker can use a dynamic expression to bypass the filters. The attacker can alternatively leverage other scripting platforms that the application server provides, such as Visual Basic (VB), to create a script that can pass through undetected firewall filters. The attacker uses NULL bytes in the second attack vector, to bypass the WAF filter. Other techniques used include event handlers, like onclick and onmouseover, which bypass signature-based WAF filters. Some WAFs limit the script length that can be inserted as payload. The length limits can also be bypassed by using a script being loaded from a remote source, e.g., <script src=http://remote-server/malicious.js></script>. Next, we describe how this process of blocking and bypassing web attacks can be formulated as a two-player zero-sum game and modeled as a GAN.

### 3.2. Generative Adversarial Networks (GANs)

A GAN defines two neural networks: generator *G* and discriminator *D* [46]. In a traditional adversarial network, the data distribution of the generator is defined as $p_{g}$ over data *x*. A prior input $p_{g}\left(z\right)$ is used as an input noise variable. The mapping of the input noise to the data space is represented as $G(z;\theta_{g})$. The generator *G* is a differential function represented by an MLP with parameter $\theta_{g}$. The second MLP used in this model, called the discriminator, is represented as $D(x;\theta_{g})$, which outputs a scalar. D(x) represents the probability that *x* came from data rather than from noise $p_{g}$.

The variable $p_{data}\left(x\right)$ refers to the original data distribution. $E_{x∼p_{data}\left(x\right)}$ is the expectation value function: it means that the expected value of x is assumed to be distributed over $p_{data}\left(x\right)$. The value function V(G,D) represents a min–max game between the generator and the discriminator. The discriminator is trained to maximize ($max_{D}$) the probability of assigning the correct label to the training example logD(x). Simultaneously, the generator is trained to minimize ($min_{G}$) the function log(1−D(G(z)). In summary, a min–max game with value function V(G,D) is defined as

(1) $$min_{G}max_{D}V\left(D,G\right)=E_{x∼p_{data}\left(x\right)}\left[logD\left(x\right)\right]+E_{z∼p_{z}\left(z\right)\left[log\left(1-D\left(G\left(z\right)\right)\right)\right]}.$$

The initial samples generated by *G* are not optimal enough to bypass the detection criterion of *D*, and are rejected by the discriminator. The generator keeps generating the adversarial samples and updating the parameters for the subsequent samples, and the generator learns a better evasion technique, to fool the discriminator.

### 3.3. GANs for Generating Web Attacks

#### 3.3.1. Motivating Example

GANs can be used to generate simulated attack data, such as malicious input payloads for injection attacks (e.g., SQL injection, XSS) or evasion techniques for bypassing security filters. These simulated attacks can be employed to evaluate the effectiveness of security mechanisms and to identify potential vulnerabilities. In this work, we improved the structure of GAN modeling, by using conditional sequencing. We considered conditional sequence generation as a process of identifying stochastic reinforcement learning policy. The policy rewards are judged on the complete sequence of the attack payload and are passed to intermediate state-action pairs, using the Monte Carlo (MC) search process.

**Figure 3:**
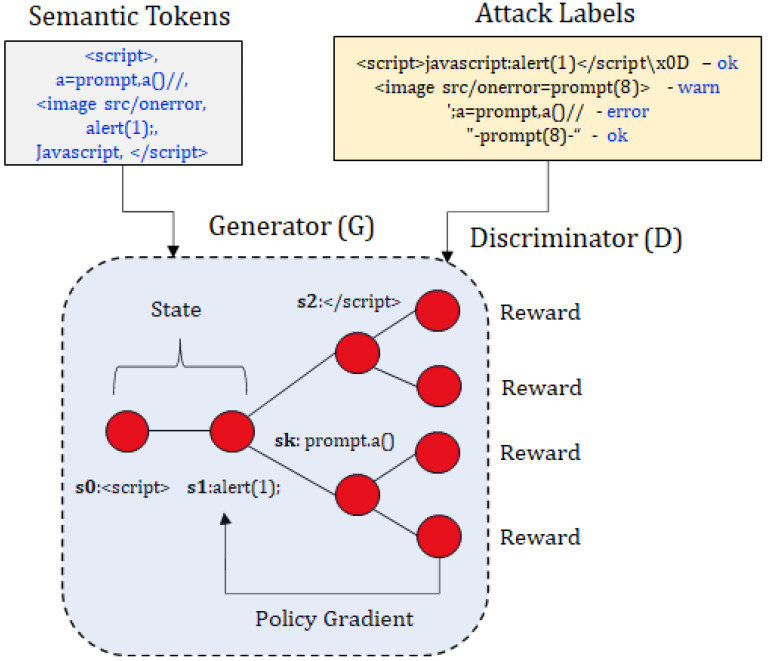
Example of conditional sequences generated from semantic tokens.

Consider Figure 3. We assume that the provided dataset has known payloads used for XSS attacks. We use a process known as semantic tokenization, which will be elaborated in Section 4.1, to obtain tokens from the initial dataset, which represents different feature values for XSS attacks, e.g., <script> is a *tag* attribute, while alert(1); is a *function body* attribute. It is difficult to label all such attributes, so we classify the attributes with no such classification by using the *other* label. Moreover, we also know that attacks provided by a dataset can be replayed against a malicious web application, to check the validity of the attack. We obtain different results when we replay these attacks against known vulnerable applications, such as DVWA, Gruyere, and OWASP vulnerable web applications. If the attack payload generates a stored, reflected, or domain-based XSS attack, we add the label *ok*. If there is an error when the payload is replayed against the web application, we add the label *error*. If nothing happens when the payload is replayed on the vulnerable web page, we add the label *fail*.

These labels and tokens are passed to the generator, *G*. Generating conditional semantic sequences starts by generating a random initial state, e.g., $s_{0}=$<script>. The next state, $s_{1}$, is selected from the list of available tokens, e.g., the action selects token alert(1); and the model transitions to state $s_{1}$. The state transition is deterministic, based on the action selected, $s_{0}\times a\mapsto s_{1}$. In this example, a = alert(1); and $s_{2}$ = <script> alert(1);. The entire sequence $\left\{s_{1},s_{2},...,s_{N}\right\}$ is evaluated by the discriminator, to check if the generated sequence is a valid attack. The discriminator is also pre-trained on both valid and invalid attack sequences, since pre-training helps improve the generator’s efficiency. Consider the generated sequence <script>alert(1);<script>: the model achieves a higher reward from the discriminator, because this sequence passes the fitness function test for a valid attack. In case the generated attack sequence is not valid, the model utilizes policy gradient and a Monte Carlo search based on the expected reward from the discriminator model.

#### 3.3.2. GAN for Bypassing a Web Application Firewall

**Figure 4:**
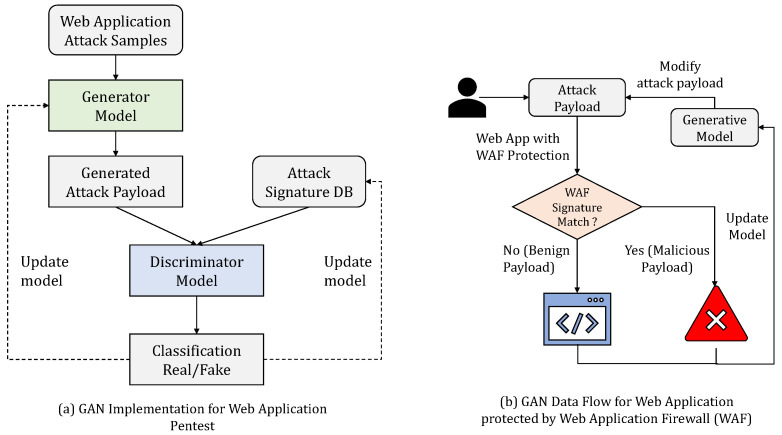
GAN-based approach for generating attack payloads that bypass web application firewall (WAF) filters.

Figure 4 provides a GAN framework for a web application pentest. The generator model uses the web application attack samples from a distribution of attack samples tried and tested as payloads. As shown in Figure 4a, the generator passes the attack payload to the discriminator. The attack sample is validated against a web application, to check if it generates an exploit against the web application. The discriminator model uses known attack samples that have worked on the application, to check if the sample provided by the generator will work on the web application. The classification result is used to classify the attack sample as valid/invalid. The model of the generator and the attack signature database are updated, based on the result.

In order to understand the semantic meaning of using a GAN against a web application and to showcase the practical application of GAN-generated payloads, consider Figure 4b, where an attack payload from a generator is replayed against a web application protected by a WAF. The WAF signature match is used as a criterion to classify an attack as malicious or benign. The attack payloads are tried against the WAF, to check if they are identified as malicious and are blocked. Using a GAN-based attack payload generation and validation mechanism, we can generate payloads that trigger web application vulnerabilities but are not classified by the WAF as malicious. During the subsequent rounds of training for the GAN, the generative model can be updated with improved versions of the attack payloads. This approach will be beneficial for generating valid attack payloads for a large-scale web application platform that is difficult to test by using known attack payloads or a manual pentesting approach. One challenge to the direct use of a GAN for non-image datasets, such as cyber-intrusion detection systems, is that features present in these datasets are discrete. Thus, numeric 0-1 features and non-numeric features are represented using One-Hot Encoding or Dummy Encoding. The dimension expansion used to account for this encoding leads to the problem of vanishing gradient [47]. Chen et al. [48] used a Wasserstein-Distance-based modified training goal to deal with the vanishing gradient problem. The research work used an additional variable Encoder (E) to train the modified GAN network. Another problem with the direct use of a GAN for generating sequential data that represent an attack such as XSS is that a GAN is designed for generating real-valued, continuous data. However, using a GAN to generate a sequence of discrete tokens is challenging. The GAN can give the score/loss for the entire sequence when it has been generated; the measure of fitness for the partially generated sequence is quite difficult. SeqGAN [23] considered sequence generation as a sequential decision making process, and the generative model was treated as an RL agent. The state was generated tokens so far, and the action was the next token in the sequence. The authors used the policy gradient method and employed an MC search to approximate state-action value. In the next section, we explain how we used a SeqGAN framework with conditional token encoding for training a GAN network and generating valid attack payloads.

## 4. Conditional Attack Sequence Generation

### 4.1. Attack Payload Tokenization

Tokenization is the process of breaking raw text into small chunks. The tokens can be groups of characters, words, or sentences. The tokens help interpret the meaning of the text, by analyzing the sequence of words (tokens). In text tokenization, the parts of the text that do not add any special meaning to the sentence, such as stop words, are removed. Removing these words from the dictionary reduces the noise and dimension of the feature set. There are different ways to perform tokenization. Some popular techniques include white-space, dictionary-based, rule-based, regular expression (regex)-based and subword-based token generation. Most of these methods suffer from inherent limitations, e.g., limitations on vocabulary size and handling words that are absent in the vocabulary. Techniques such as BPE are used to deal with the Out-Of-Vocabulary (OOV) sequences. It segments OOV as subwords and represents the words in terms of those subwords.

The tokenization method that identifies meaningful attack payload tokens can be applied to a dataset of attack inputs, such as XSS, to identify relevant sub-sequences that can be combined to target the vulnerable web application. Semantic tokenization uses markers (such as tags *<>, <script*), parameter names (such as *href=*), function body (such as *alert(*), common words (such as *javascript, VBScript*), and special encoding (such as *u003c*), http/https links. Once the semantic meaning has been assigned to the tokens, the BPE [25], a variant of Huffman Encoding, is applied to the semantically labeled tokens. It uses more embedding or symbols for representing less frequent terms in the corpus.

**Figure 5:**
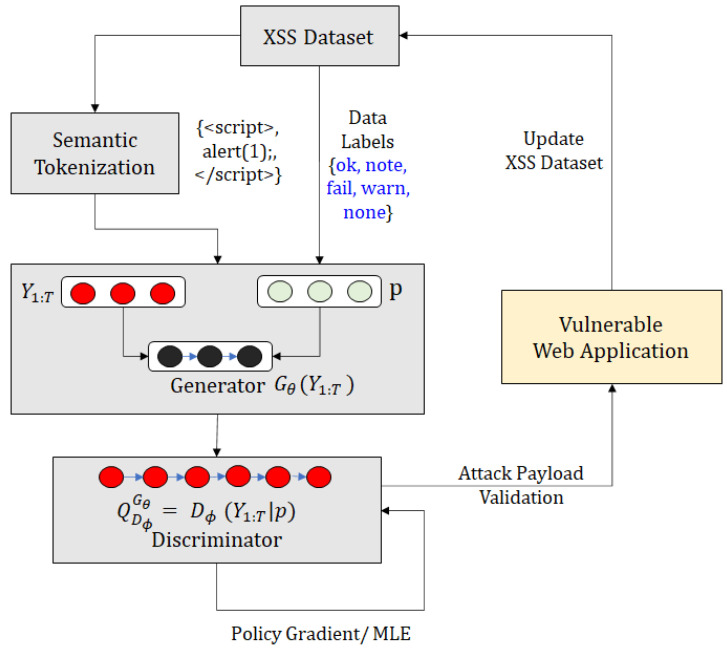
Conditional Attack Sequence Generation by semantic tokenization and attack payload validation.

The semantic tokenization process takes the XSS dataset as input, as shown in Figure 5. The input text is split, based on matching conditions for the markers, such as tags, encoding, function body, and parameter name. The input corpus (XSS dataset) is parsed line by line, and the data are converted to the HTML-rendered format. The rendered data from each are replayed against vulnerable applications. We utilized *Burpsuite* [49] to replay the initial attack data *D* and to label each attack payload, based on the result of the HTML code replayed against the web application (ok, note, warn, fail, error). The labels were used as input for the conditional sequential GAN. The tokens from each line were annotated and grouped into frequently occurring symbols, using a BPE algorithm. These were added to the vocabulary of the known tokens. The process was repeated until no new combination of symbols was present. The vocabulary and data labels were passed to the generator, *G*.

### 4.2. Conditional Sequencing

The architecture for conditional attack sequence generation has been described in Figure 5. We use the input data from the XSS dataset $D_{a}$. The data are preprocessed, to extract the semantic tokens. The XSS data are also labeled with the results of attack sequences that are replayed on a vulnerable web application. The label information *p* and tokens $Y_{1:T}$ are passed to the generator, $G_{\theta }\left(Y_{1:T}\right)$. The discriminator is assumed to have inputs from the original dataset $\chi_{1:N}$ and from some fake data generated from input sequences that failed to generate valid alerts on vulnerable web applications. The discriminator $D_{\phi }\left(Y_{1:T},p\right)$ utilizes the policy gradient or the Maximum Likelihood Estimate (MLE) for learning optimal policies for the generation of attack sequences, $Q_{D_{\phi }}^{G_{\theta }}$. The attack payloads are validated against vulnerable web applications, and the payloads that pass the validation phase are added to the original XSS dataset $D_{a}$.

We consider the dataset $\chi_{1:N}$ and the labeling information *p* as the initial input to the sequence generation process. The generative model is θ-parameterized, and the model parameters can be determined by the data distribution based on labels *p*. The goal of generator $G_{\theta }$ is to produce a sequence $Y_{1:T}=\left\{y_{1},y_{2},\cdots ,y_{T}\right\}$, such that $y_{t}\in Y$, where Y is the vocabulary of the candidate tokens extracted from $\chi_{1:N}$. This process can be considered an RL policy generation problem. The policy generation process is a modified version of sequence generation, as discussed in SeqGAN [23], with semantic tokenization and conditional labeling. The policy model for conditional sequencing $G_{\theta }\left(y_{t}|Y_{1:t-1},p\right)$ is stochastic. The transition between states is deterministic, i.e., $\delta_{s,s^{\prime }}^{a}=1$, where $s=Y_{1:t-1}$, $s^{\prime }=Y_{1:t}$, $a=y_{t}$, and, for all other states, $s^{\prime \prime }$. The discriminator model $D_{\phi }$ is ϕ-parameterized for improving the generator, $G_{\theta }$. The model $D_{\phi }\left(Y_{1:T}|p\right)$ is a problem indicating how likely the sequence is, from the real attack dataset $D_{a}$. The discriminator is trained by providing positive examples from the attack dataset $D_{a}$ and negative examples from the synthetic dataset. The negative examples are malformed attack payloads that fail the XSS attack test on vulnerable web applications.

#### Conditional Sequence-Based Attack Generation

The objective of the generator model $G_{\theta }\left(y_{t}|Y_{1:t-1},p\right)$ is to generate a sequence from the start state $s_{0}$, the model parameters θ, and the attack labels *p*. As an example, the start state could be one of the semantically labeled tokens, e.g., $s_{0}$ = </scrip</script>t>. The goal of the model is to maximize the expected reward $R_{T}$ for the generation of a complete attack sequence, described by Equation (2). The function $E[R_{T}|s_{0},\theta ,p]$ represents the expected reward, given the labels, start state, and model parameters:

(2) $$J\left(\theta \right)=E\left[R_{T}|s_{0},\theta ,p\right]=\sum_{y\in Y}G_{\theta }\left(y_{1}|s_{0},p\right).Q_{D_{\phi }}^{G_{\theta }}\left(s_{0},y_{1}\right).$$

The $Q_{D_{\phi }}^{G_{\theta }}\left(s,a\right)$ is the action value function for a sequence, i.e., the expected reward accumulated by starting with the initial state *s*, taking the action *a*, and following the conditional sequence $G_{\theta }$ parameterized by the attack labels. The objective function for the sequence starts from the initial states. It follows the policy to generate a sequence of tokens $Y_{1:T}=\left\{y_{1},..,y_{t},..y_{T}\right\}$ that can be considered real attacks when evaluated on the vulnerable web application—Figure 5. The action-value function REINFORCE [50] is used by the discriminator $D_{\phi }\left(Y_{1:T}^{n}\right)$ for estimating the reward. The reward calculated by the discriminator is for the finished attack sequence. The model captures the fitness of the previous tokens in the attack sequence (prefix) and the resulting future outcomes. The model utilizes an MC search with the roll-out policy $G_{\beta }$, to sample T−t unknown tokens. The N-time MC search procedure is represented by Equation (3) below:

(3) $$\left\{Y_{1:T}^{1},\cdots ,Y_{1:T}^{N}\right\}=MC^{G_{\beta }}\left(Y_{1:t};N|p\right).$$

The tokens are $Y_{1:t}^{n}=\left(y_{1},\cdots ,y_{t}\right)$, and $Y_{t+1:T}^{n}$ is sampled, based on the roll-out policy $G_{\beta }$ and the current state. The roll-out policy is started from the current state, and run for N times, to obtain the batch output of the attack samples. The roll-out policy for the conditional sequence starts from the current state and runs till the end of the sequence, for N times, to obtain a batch of output samples. As described in Equation (4), this process reduces the variance and obtains a more accurate assessment of the action value:

(4) $$\begin{array}{c} Q_{D_{\phi }}^{G_{\theta }}\left(s=Y_{1:t-1},a=y_{t}|p\right)=\frac{1}{N}\sum_{n=1}^{N}D_{\phi }\left(Y_{1:T}^{n}|p\right),\\ Y_{1:T}^{n}\in MC^{G_{\beta }}\left(Y_{1:t};N|p\right)\;for\;t<T\\ Q_{D_{\phi }}^{G_{\theta }}\left(s=Y_{1:t-1},a=y_{t}|p\right)=D_{\phi }\left(Y_{1:t}|p\right)\;for\;t=T \end{array}$$

The process does not provide intermediate rewards; instead, the function iteratively updates and improves the generative model, starting from $s^{\prime }=Y_{1:t}$. The discriminator is retrained when more realistic attack payloads are generated from the model. In turn, the new discriminator model is used to retrain the generator. The policy-based model optimizes the parameterized policy, to maximize long-term rewards directly.

**Figure 6:**
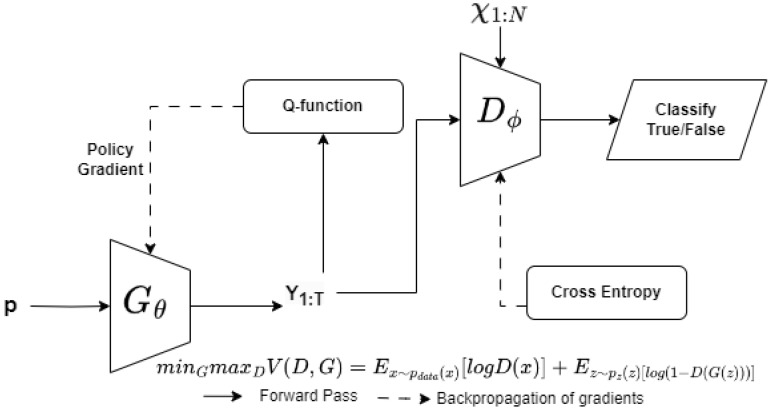
Conditional Attack Sequence Generation.

We describe conditional sequence generation and XSS attack test procedures in Figure 6, and we also provide the detailed Algorithm 1, for the same. The generator $G_{\theta }$, parameterized by attack labels *p*, is pre-trained on *S*, using the MLE algorithm. The supervised signal from the pre-trained discriminator helps improve the generator’s efficiency. The generator is conditioned on the attack labels *p* and trained for *g*-steps, to generate the sequence $Y_{1:T}$ (line 6). The Q-function $Q_{D_{\phi }}^{G_{\theta }}$ is calculated for each step of the generator (line 7). If the current state is represented by $s=Y_{1:t-1}$ and the action is $a=y_{t}$, the next state is calculated by using the action-value function. The generator is updated, using the policy gradient approach described earlier. The discriminator needs to be re-trained periodically, to improve its performance. The positive examples are provided from training set *S*, and the negative examples are provided from the failed attack sequences from the generator. The number of positive and negative examples is the same for each d-step in the algorithm. The trained generator is used for XSS attack validation, by replaying the sequences against vulnerable web application lines 18–24. The valid attacks are added to the base initial training set $\chi_{1:N}$, to improve the variability of the training data.
**Algorithm 1** Conditional Sequence Generation1:**procedure** Conditional Sequence Generation($X_{1:N},p$)2:    Initialize $G_{\theta }$, $D_{\phi }$, p, β←θ3:    $G_{\theta }$ pre-trained using MLE on *S*, p4:    Train $D_{\phi }$ from positive, negative $G_{\theta }$ samples5:    Pre-train $D_{\phi }$ to minimize cross entropy6:    **for** g-steps **do** Generate $Y_{1:T}=\left(y_{1},..,y_{T}|p\right)∼G_{\theta }$7:        **for** t ∈ {1:T} **do**8:           Calculate Q-function $Q(a=y_{t};s=Y_{1:t-1}|p)$9:        **end for**10:      Update generator using policy gradient11:    **end for**12:    **for** d-steps **do**13:        Generate true alerts, false alerts using $G_{\theta }$, *S*14:        Train $D_{\phi }$ for *k*-epochs15:    **end for**16:**end procedure**17:**procedure** XSS Attack Test($G_{\theta },S,\chi_{1:N}$)18:    **for** s ∈*S*, $G_{\theta }$ **do**19:        s ← html_render(s)20:        **if** xss_eval (s) **then**21:           assign_label (s)22:           update $\chi_{1:N}$, add *s*23:        **end if**24:    **end for**25:**end procedure**

## 5. Experimental Evaluation

We used the XSS dataset [51] collected from multiple XSS scanning tools containing the payload data covering different XSS attacks. The dataset covers different features of XSS attacks, such as tags, function body, URL, and encoding.

### 5.1. Evaluation of Loss for Conditional GANs

**Figure 7:**
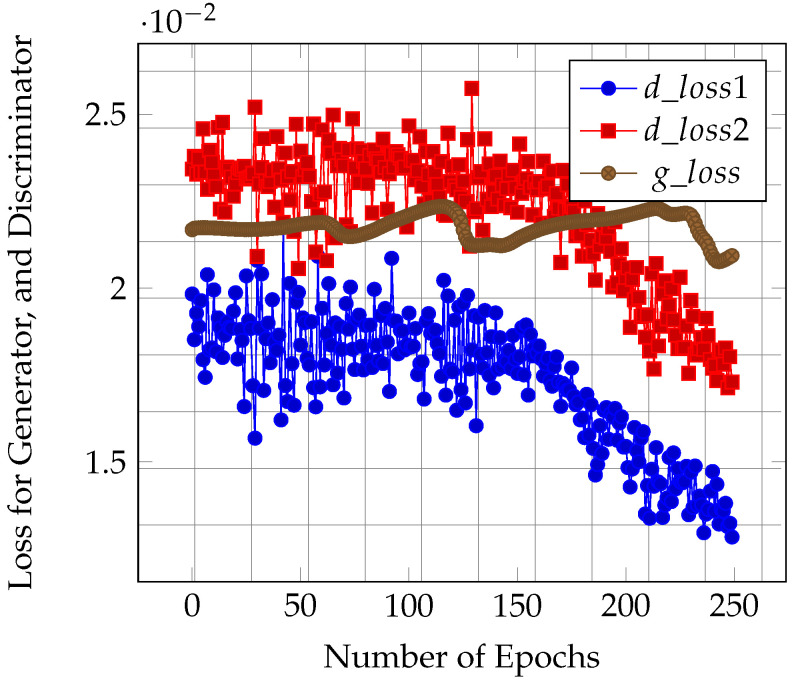
The result of GAN training loss for 250 epochs.

We utilized the sample payloads from the XSS dataset [51] to train our GAN model. The discriminator loss consisted of two parts, i.e., d_loss1 and d_loss2. The first loss value detected real attack samples as real, and the second loss detected fake attack samples as fake. On the other hand, the generator loss tried to generate attack samples that were hard for discriminators to detect as real attacks. The generator and discriminators were trained to improve loss functions till convergence was achieved. We observed that our discriminator loss functions decreased as the number of training samples increased, converging to a stable value ∼1.1 $\times \;10^{-2}$ (see Figure 7). This meant that our discriminator was more accurate at distinguishing between real and fake attack samples. The value of the loss function for the generator also decreased with time, reaching a minimum value of ∼250 epochs. We observed that there was no further improvement in the loss function of the discriminator. This signified an improvement in the quality of generating attack samples that could fool the discriminator’s ability to detect attack payloads. In summary, the attack samples generated at around 250 epochs could be utilized to test the web application firewall’s effectiveness in detecting attacks.

### 5.2. Web Application Firewall Bypass

#### 5.2.1. ModSecurity WAF Testing

We utilized the ModSecurity WAF to check the attack payloads generated by different variants of the GAN network. The attacks were first verified over vulnerable web applications and were then replayed against the WAF, to check how many attacks were detected by the rules of the WAF. ModSecurity consists of modules, such as PhantomJS (a headless WebKit with JavaScript API). The module uses WebKit’s browser environment to detect reflected XSS attacks accurately. For instance, XSS attack payloads use partial non-alphanumeric obfuscation. The code

<script>eval("aler"+(!![]+[])[+[]])("xss")</script>

can be used to bypass normal XSS detection filters. The PhantomJS conducts execution time analysis within the browser Document Object Model (DOM) after de-obfuscation, to validate the attack payload. Other modules, such as Lua API, allow the security team to hook in external programs that extract HTTP data and pass it to PhantomJS for detection.

We evaluated the effectiveness of a vanilla GAN and a conditional GAN (CGAN) on a vulnerable web application protected by a ModSecurity WAF. For each trial run in Table 1, we randomly selected payloads from the XSS dataset. The test set consisted of payloads generated by the vanilla GAN and the CGAN. The percentages represent the success rate for each version of the GANs, i.e., the number of valid attack samples that were generated after the training of the GAN was finished. These payloads were able to successfully bypass the WAF. During the experiment, we found that 10.37% of the vanilla GAN payloads could bypass the ModSecurity WAF filters in the first batch, whereas for the CGAN only 7.66% of the generated payloads could bypass the WAF. For the second batch, the CGAN performed slightly better than the vanilla GAN (see Table 1). We observed good performance for the CGAN in the fourth batch, i.e., only 0.08% of the vanilla GAN payloads were able to bypass the WAF, whereas 12% of the CGAN payloads were able to bypass the WAF. This meant that the CGAN was more consistent in providing valid payloads across all the trial runs. This was because the CGAN utilized semantic tokenization to understand the structure of the XSS payloads and mimicked the valid payloads closely. In effect, the quality of generated attack samples was better for the CGAN.

#### 5.2.2. AWS WAF Testing

We enabled the AWS WAF to protect a commercial-grade web application. The infrastructure in AWS requires the creation of an application load balancer (ALB) or an API gateway. We utilized an ALB for our setup [52]. We created a custom ruleset, including AWS pre-set rules, to detect and prevent attacks, like URI path inclusion, SQLI, anonymous IP address, and different variants of XSS attacks. The commercial rules from AWS marketplace vendors like Fortinet and F5 were also used as a part of the AWS access control list (ACL). The ACL was attached to the created ALB. In total, the WAF comprised 3000 rules. The attack payloads generated using the conditional GAN were replayed against the AWS WAF, and the results were observed in the AWS WAF management dashboard.

The % Attack Match means the percentage of valid payloads. We observed that 8% of the GAN-generated payloads were able to bypass the rulesets of the AWS WAF (Table 2). This means that the success rate for the CGAN on commercial firewalls is quite low. This was expected, because commercial firewalls utilize a broader set of signatures to detect web attacks. We observed that 44.9% of attack payloads were detected under AWS-managed XSS rules, whereas 44% of payloads were detected using Fortinet-based commercial rules downloaded from the AWS marketplace. The WAF misclassified 3.1% of the attack payloads as SQLI attacks. This indicates that attack signatures from commercial WAFs are prone to minor errors. While the bypass rate was quite low for the AWS WAF, a malicious attack group only requires a few valid signatures to bypass a WAF; thus, security teams can utilize the valid attack signatures to update the WAF rulesets.

/?saivs.js%20%20/%3E%3Cvideo%3E%3Csource%20%20/%3E

/?%3Ca/src=/%20%3C/img%3E%3Cinput%20’

/?%5Cu0061lert%60%60;

An example of attack payloads that successfully bypassed the AWS WAF can be seen above. The caveat that attackers use to bypass XSS filters includes removing different parts of the attack string and checking if the input is still blocked. Other methods include using alternate means of introducing scripts, such as img tags, event handlers, script pseudo-protocols (such as javascript), and dynamically evaluated styles. The discriminator in a GAN learns these variations after being trained on attack payloads, and, hence, the discriminator can generate attack sequences with no matching attack signatures.

### 5.3. Comparative Analysis with Existing Research

Our research provides a unique mechanism for learning the signatures configured in a given WAF and new data that can be used for improving attack detection systems. We identified several gaps in the existing research on autonomous web app pentesting. We identified that, except for the POMDP+ model proposed by Schwartz et al. [33], most research fails to incorporate the defender’s perspective when modeling autonomous pentesting. Our GAN model captures the attacker’s and the defender’s perspective through generator and discriminator models. Some other limitations of the existing research include the use of static configurations [28], lack of practical evaluation [34], and showing limited scalability on an extensive network [35]. We compared the convergence rate of a CGAN network to the POMDP model proposed by Schwartz, et al. [31]. The authors conducted training on single and multi-site networks, and convergence took ∼1000 episodes, which was 4× that of the convergence achieved by conditional GAN network in the proposed solution. This is due to a better understanding of attack semantics in our model.

## 6. Discussion

We discussed the challenges posed by integrating cloud computing with WSNs and incorporating the IoT in these networks. Although there are defense mechanisms, such as commercial WAFs, to identify these security issues, the sophistication of these attacks keeps increasing, including deceptive means used by attackers to masquerade as genuine traffic. Attacks such as APTs require the identification of attack patterns beyond traditional signature-based detection. Penetration testing plays a crucial role in identifying security issues and risks related to the IoT, sensor networks, smart solutions, and web-based vulnerabilities. A significant shortage of cybersecurity professionals has led to a demand for AI-enabled penetration testing techniques.

GAN provides a mechanism to mimic a pentester and a network defender in a two-player zero-sum game. Using GANs to generate discrete data or attack sequences, such as XSS and SQL Injection payloads, is challenging. The generation process can suffer from inherent limitations, such as poor input quality, lack of diversity, and mode collapse, as discussed in SentiGAN [53]. We propose a conditional sequence generation mechanism that utilizes the pentester’s semantic knowledge in the generative model for performing autonomous pentesting against commercial WAFs, such as ModSecurity and AWS WAF.

The practicality of the proposed approach was highlighted by learning the success rate of valid attack payloads on WAFs. Our model achieved fast convergence, due to the semantic encoding of attack tokens. Moreover, the synthetic data generated by the generator upon convergence can help to improve the signature database of the WAFs. Although we achieved some valid payloads, the sequence GAN framework used in our work suffered from the inherent limitation of capturing long-term dependencies between sequences—hence, performing poorly on an AWS WAF. Category-aware generative networks with hierarchical learning models [54] could help overcome some of the limitations present in sequence GANs.

## 7. Conclusions and Future Work

We propose a GAN-based solution for modeling web application attacks and conducting autonomous pentesting on applications protected by commercial WAFs. Our model utilizes conditional sequence generation, to learn the structure of attack payloads that can bypass WAFs. Experimental evaluation conducted on a ModSecurity WAF and an AWS WAF generated several valid payloads. Our proposed solution tests the rule structure of WAFs, by generating new payloads that are hard to detect, using existing WAF rules. These payloads can, in turn, be utilized to improve the robustness of WAFs and to deal with sophisticated attacks. A limitation of our work is the low bypass rate on commercial-grade WAFs, such as the AWS WAF. We plan to explore alternate GAN versions, to better model the long-term sequences of valid attack payloads. This approach could help to improve our model’s performance on commercial WAFs. We also plan to expand our experimental section attacks, such as CSRF, SQLI, and directory traversal attacks. 

## Figures and Tables

**Table 1:** Number of successful WAF bypasses, using several variants of GAN.

Run #	Vanilla GAN	CGAN
1	10.37%	7.66%
2	7.69%	8.04%
3	17.64%	9.08%
4	0.08%	12%
5	16.19%	8.28%

**Table 2:** GAN-generated XSS attack payloads against AWS WAF.

Matching Rule	% Attack Match	AWS WAF Action
WAF Bypass	8.0%	ALLOW
AWS-managed XSS	44.9%	BLOCK
Fortinet XSS Rule	44.0%	BLOCK
Misclassified	3.1%	BLOCK

## Data Availability

Data available in a publicly accessible repository that does not issue DOIs Publicly available datasets were analyzed in this study. This data can be found here: https://github.com/payloadbox/xss-payload-list.
